# Supplementary material for: Effect of Low-Level Tragus Stimulation on Cardiac Metabolism in Heart Failure with Preserved Ejection Fraction: A Transcriptomics-Based Analysis
Source: Int J Mol Sci. 2024 Apr 13;25(8):4312. doi: 10.3390/ijms25084312 (PMC11050145; doi:10.3390/ijms25084312)
Supplement: Supplementary file 1 [file ijms-25-04312-s001.zip › ijms-2965501-supplementary.pdf]

SUPPLEMENTAL MATERIALS

Effect of low-level tragus stimulation on cardiac metabolism in heart failure with preserved ejection fraction: A transcriptomics-based analysis

Chakraborty *et al*.

SUPPLEMENTAL TABLES

Table S1: Significantly altered canonical pathways in HFpEF (LS vs HS-sham)

Canonical pathway	-log(p-value)	z-score	DEGs
Oxidative Phosphorylation	14.4	-4.707	ATP5MF, ATP5PB, ATP5PO, COX10, COX5B, COX6A2, Cox6c, ATP5PD, COX7A2L, NDUFA11, NDUFA4, NDUFA5, NDUFAB1, NDUFB10, NDUFB3, NDUFB4, NDUFB5, NDUFB8, NDUFS1, NDUFS4, NDUFS6, NDUFS8, NDUFV2, UQCRC1, UQCRC2, UQCRQ
Fatty Acid β-oxidation I	6.02	-3	ACAA2, ACSL1, ECHS1, ECI1, HADH, HADHA, HADHB, HSD17B8, SLC27A1
Valine Degradation I	8.33	-3	ALDH6A1, BCAT2, BCKDHA, BCKDHB, DBT, ECHS1, HADHA, HADHB, HIBADH
TCA Cycle II (Eukaryotic)	4.91	-2.646	ACO2, CS, DLST, FH, IDH3A, OGDH, SDHAF4
Tryptophan Degradation III (Eukaryotic)	5.05	-2.449	ACAA2, ACAT1, GCDH, HADH, HADHA, HADHB, HSD17B8
Glutaryl-CoA Degradation	6.29	-2.449	ACAA2, ACAT1, GCDH, HADH, HADHA, HADHB, HSD17B8
Stearate Biosynthesis I (Animals)	2.9	-2.236	ACOT2, ACSL1, DBT, GNPAT, PTGR2, SLC27A1, TECR
Isoleucine Degradation I	4.99	-2.236	ACAA2, ACAT1, BCAT2, ECHS1, HADHA, HADHB
PPARα/RXRα Activation	1.81	-2.496	ACADL, ACOX1, ADCY5, ADIPOR1*, GHR, GOT2, LPL, PRKACA, RAP1B, RRAS*, RXRA, SLC27A1, TGFB2*
Superpathway of Geranylgeranyldiphosphate Biosynthesis I (via Mevalonate)	3.53	-2	ACAA2,ACAT1,COX10,HADHA,HADHB
Sirtuin Signaling Pathway	10.4	3.9	ACADL, ATP5PB, BAX*, BECN1*, BPGM*, GADD45A*, GOT2, H3-3A/H33B*, LDHD, MAP1LC3B2*, NDRG1*, NDUFA11, NDUFA4, NDUFA5, NDUFAB1, NDUFB10, NDUFB3, NDUFB4, NDUFB5, NDUFB8, NDUFS1, NDUFS4, NDUFS6, NDUFS8, NDUFV2, PDK1, PGAM1*, PGAM2, Polr1d*, SLC25A4, STAT3*, TIMM50, TUBA1C*, TUBA8, UQCRC2, VDAC1

*: Upregulated genes

Table S2: Effect of LLTS on canonical metabolic pathways (HS-active vs HS-sham)

Canonical pathway	-log(p-value)	z-score	DEGs
Oxidative Phosphorylation	8.05	-2.309	Atp5e, ATP5F1D, ATP5MC3, ATP5PB, Cox6c, COX8A, NDUFA12, NDUFB7, NDUFB9, NDUFS3, NDUFS7, UQCRFS1

Table S3: Top 15 inhibited upstream modulators in HFpEF (LS vs HS-sham)

Rank	Upstream Regulator	p-value	z-score	Target Molecules
1.	PPARGC1A	2.8E-22	-5.756	ACACB, ACADL, ACADVL, ACAT1, ACOX1, ATP5MF, ATP5PD, ATP5PO, BAX, BCAT2
2.	INSR	1.16E-24	-5.55	ACAA2, ACADL, ACADVL, ACO2, ACOT13, ACOT2, ACSL1, ACTA1, ALDH6A1, ANKRD1
3.	Alpha catenin	9.11E-09	-4.007	BCL2L1, COL1A1, COL1A2, COL3A1, CTSB, CTSV, ELN, FSTL1, IGFBP4, IGFBP6
4.	TSC2	5.91E-12	-3.981	ANXA1, ANXA2, ATF4, ATP6V0B, BAX, CCND3, CDKN1A, CREG1, CRYAB, DDIT3
5.	KLF15	9.52E-16	-3.897	ACADL, ACADVL, ACAT1, ACOX1, ACSL1, ACSS1, BECN1, CCN2, CPT2, FABP3
6.	PPARA	2.21E-16	-3.778	ACAA2, ACADL, ACADVL, ACAT1, ACOT2, ACOX1, ACSL1, ACTA1, ALDH9A1, ANXA2
7.	HBA1/HBA2	1.85E-10	-3.742	ATP5MF, ATP5PO, COX6A2, Cox6c, NDUFA11, NDUFA4, NDUFA5, NDUFB4, NDUFB8, NDUFS4
8.	miR-199a-5p (and other miRNAs w/seed CCAGUGU)	0.000000326	-3.69	ACTA1, ACTG2, ALOX5AP, CDKN1A, COL1A1, COL4A1, CSTB, FN1, ID3, ISG15
9.	miR-30c-5p (and other miRNAs w/seed GUAAACA)	0.0000372	-3.532	ATP2A2, BECN1, CCN2, COL1A1, COL1A2, COL4A1, COL8A1, GNAI2, IDH1, MLLT11
10.	PNPLA2	0.000000017	-3.516	ACAA2, ACADL, ACADVL, ACO2, ACOT2, ACOX1, ACSL1, CPT2, CS, FH
11.	KLF11	0.0000528	-3.464	CCN2, COL1A2, COL3A1, CPT2, FABP3, HADHB, IDH3A, ILK, PLAT, SERPINE1
12,	CR1L	9.65E-09	-3.278	CCN2, COL18A1, COL1A1, COL1A2, COL3A1, COL4A1, COL8A1, FBN1, MGP, SERPINE1
13.	PPARD	1.66E-12	-3.236	ACAA2, ACACB, ACADL, ACADVL, ACOX1, ALDH9A1, AQP7, BCL2L1, CPT2, ECH1
14.	let-7a-5p (and other miRNAs w/seed GAGGUAG)	0.0000355	-3.22	BCL2L1, CAPG, CHMP2A, COL1A1, COL1A2, COL3A1, COL4A1, COL8A1, GYS1, HBA1/HBA2
15.	PPARGC1B	0.000000192	-3.074	ACACB, ACADL, ACADVL, ATP5MF, ATP5PO, CIDEA, CKB, CS, MYH6, NDUFV2

Table S4: Top 15 activated upstream modulators in HFpEF (LS vs HS-sham)

Rank	Upstream Regulator	p-value	z-score	Target Molecules
1.	TGFB1	6.01E-25	6.569	ABCF1, ACAA2, ACSS1, ACTA1, ACTG2, ADAM19, ADK, ALOX15, ALOX5AP, ANKRD1
2.	TP53	1.22E-55	5.422	ACAA2, ACACB, ACADL, ACADVL, ACAT1, ACO2, ACSF2, ACSF3, ACSL1, ACSS1
3.	MTPN	4.06E-17	4.93	ANXA1, BAX, BCL2L1, BNIP3, Calm1 (includes others), CASQ1, CCND3, COL1A1, COL1A2, COL8A1
4.	CLPP	3.37E-21	4.707	ACADL, ACADVL, ACO2, ACOT13, ACSL1, CRAT, CS, DLST, ECH1, ECI1
5.	AGT	1.65E-14	4.324	ACTA1, ADIPOR1, ALOX15, ANXA1, ATP1B1, BAX, BECN1, BOK, CCN2, CCND3
6.	MAP4K4	8.47E-19	4.139	ACAA2, ACACB, ACADVL, ACO2, ATP6V0E1, BCAT2, BCKDHB, Calm1 (includes others), COQ3, COX10
7.	TNF	1.06E-18	4.13	ACADVL, ACOX1, ACSL1, ACTA1, ADCY5, AEBP1, AKR1B1, ALOX5AP, ANXA1, ANXA11
8.	MRTFB	9.5E-12	3.865	ACTA1, ANKRD1, BCL2L1, CCN2, CD151, COL3A1, CYBA, FIBIN, FILIP1L, GADD45A
9.	NRG1	1.16E-19	3.703	ACAT1, ANKRD1, ATF4, BCL2L1, BNIP3, CCN2, CCND3, CDKN1A, CHCHD3, COL1A1
10.	SLC27A2	3.21E-14	3.674	ACAA2, ACADL, ACOX1, ACSL1, ALDH1A1, CPT2, CRAT, DHRS4, ECI1, ETFDH
11.	MRTFA	5.67E-14	3.653	ACTA1, ANKRD1, ARPC1B, BCL2L1, CCN2, CCN5, CD151, CYBA, FABP3, FIBIN
12.	PRL	1.21E-23	3.609	ACTR3, ANXA2, ANXA5, ATP2A2, ATP5PD, BAG1, BCL2L1, BOK, CCND3, CDKN1A
13.	KDM5A	1.62E-15	3.528	ACO2, ACTN2, ATP1B1, BCKDHA, CHCHD3, COL1A2, DLST, DMPK, GADD45GIP1, HMOX1
14.	RAF1	3.61E-09	3.477	ANKRD1, ANXA1, BECN1, BTG1, CA2, CCN5, CDKN1A, CRYAB, EMP1, GRN
15.	IL6	0.0000135	3.441	ACOX1, ANXA1, ATP2A2, BAX, BCL2L1, CCN2, CDKN1A, CEBPD, CLU, COL1A1

Table S5: Significantly altered upstream modulators by LLTS

Rank	Upstream Regulator	p-value	z-score*	Target Molecules
1.	MAPK1	0.00343	-2.345	APOE, CAV3, CCL5, FLNC, GBP1, HBA1/HBA2, IFITM3, IRF7, JUND, PDLIM3, SERPINE1, SOCS2
2.	Gsk3	0.0032	-2.224	ATF4, DDIT3, NKX2-5, TNNC1, TNNI3
3.	RB1	0.00038	-2.21	ATP6V1F, CKM, DDIT3, DYNLRB1, GRPEL1, IFITM3, MDH1, MRPL12, NDUFS7, NKX2-5, PARK7, SERPINE1, TNNC1, TNNT2, Tpm1, UQCRFS1
4.	INSR	0.0163	-2	ATP5F1D, ATP5MC3, Calm1 (includes others), ETFB, FLNC, IFITM2, NDUFB9, SERPINE1, SIRT2, SOCS2, YEATS4
5.	IFNA2	0.0112	2.795	B2M, CCL5, GBP1, IFITM2, IFITM3, IRF7, LY6E, SOCS2
6.	STAT1	0.0168	2.764	APOE, B2M, CCL5, CLIC5, FCER1G, GBP1, IFITM2, IFITM3, IRF7, LY6E
7.	KDM5A	0.00000529	2.53	ATP6V1F, GRPEL1, MDH1, MRPL12, NDUFS7, PARK7, TNNC1, TNNT2, Tpm1, UQCRFS1
8.	IFNAR1	0.0464	2.179	B2M, CCL5, CLIC5, IRF7, SERPINE1
9.	RICTOR	1.12E-13	2.041	Atp5e, ATP5F1D, ATP5MC3, ATP5PB, ATP6V0B, ATP6V1F, Cox6c, COX8A, NDUFB7, NDUFB9, NDUFC1, NDUFS3, NDUFS7, POMP, PSMB4, PSMB5, PXN, RPL17, RPL18, RPL26, Rpl29

*: activation pattern in HS sham group compared to HS-active group

SUPPLEMENTAL FIGURES

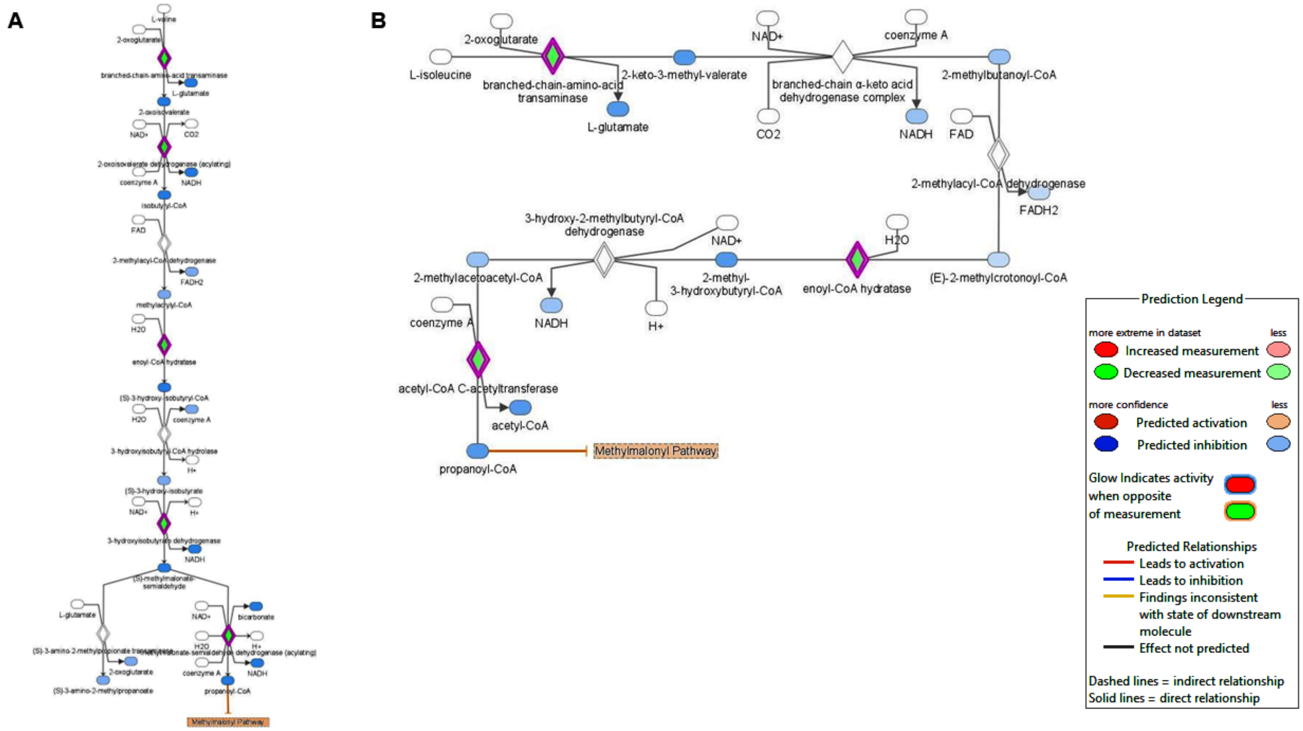

Figure S1: IPA prediction of branch chain amino acid catabolism in HFpEF (LS vs HS-sham). Valine (A) and isoleucine (B catabolism were predicted to be inhibited.

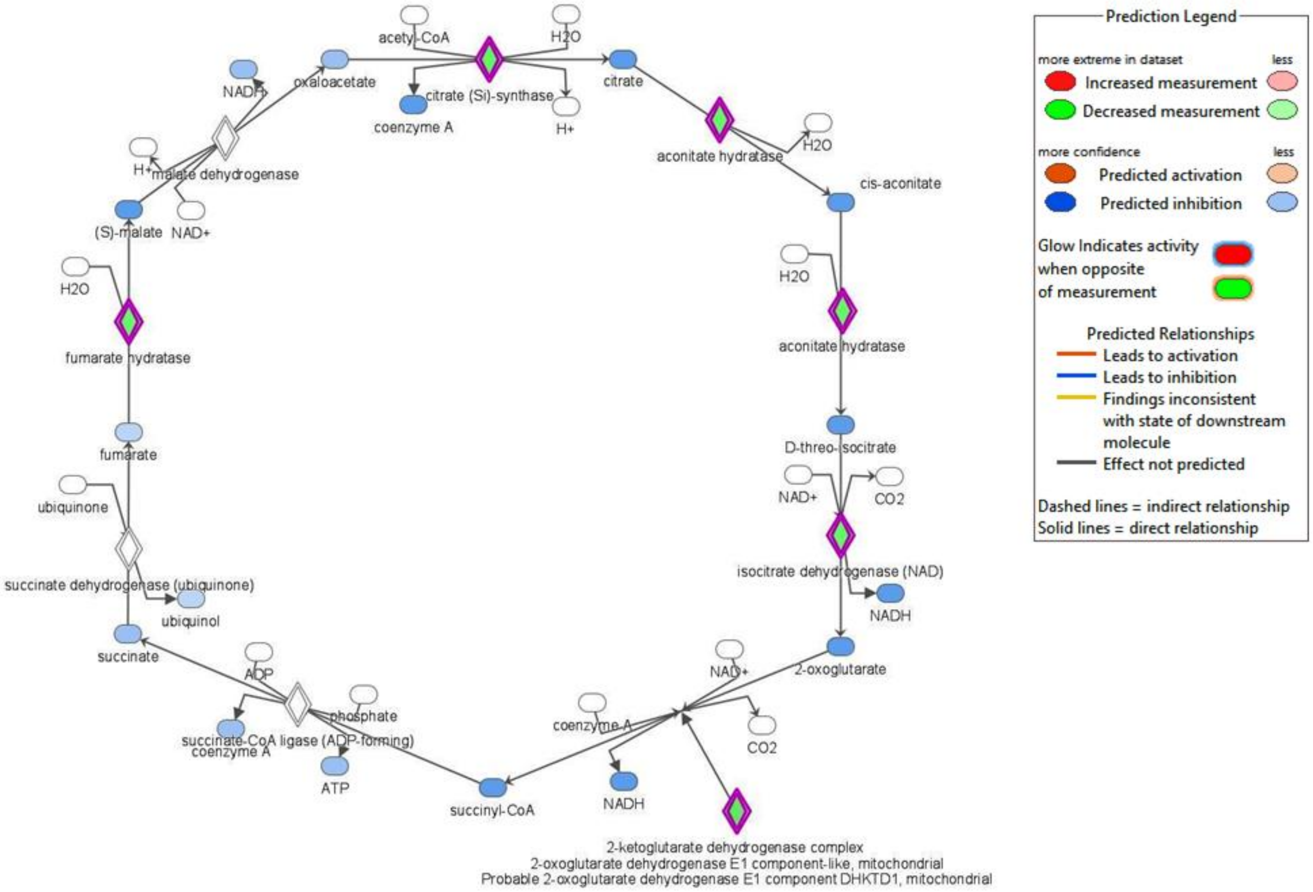

Figure S2: IPA prediction of Tricarboxylic acid cycle in HFpEF (LS vs HS-sham)

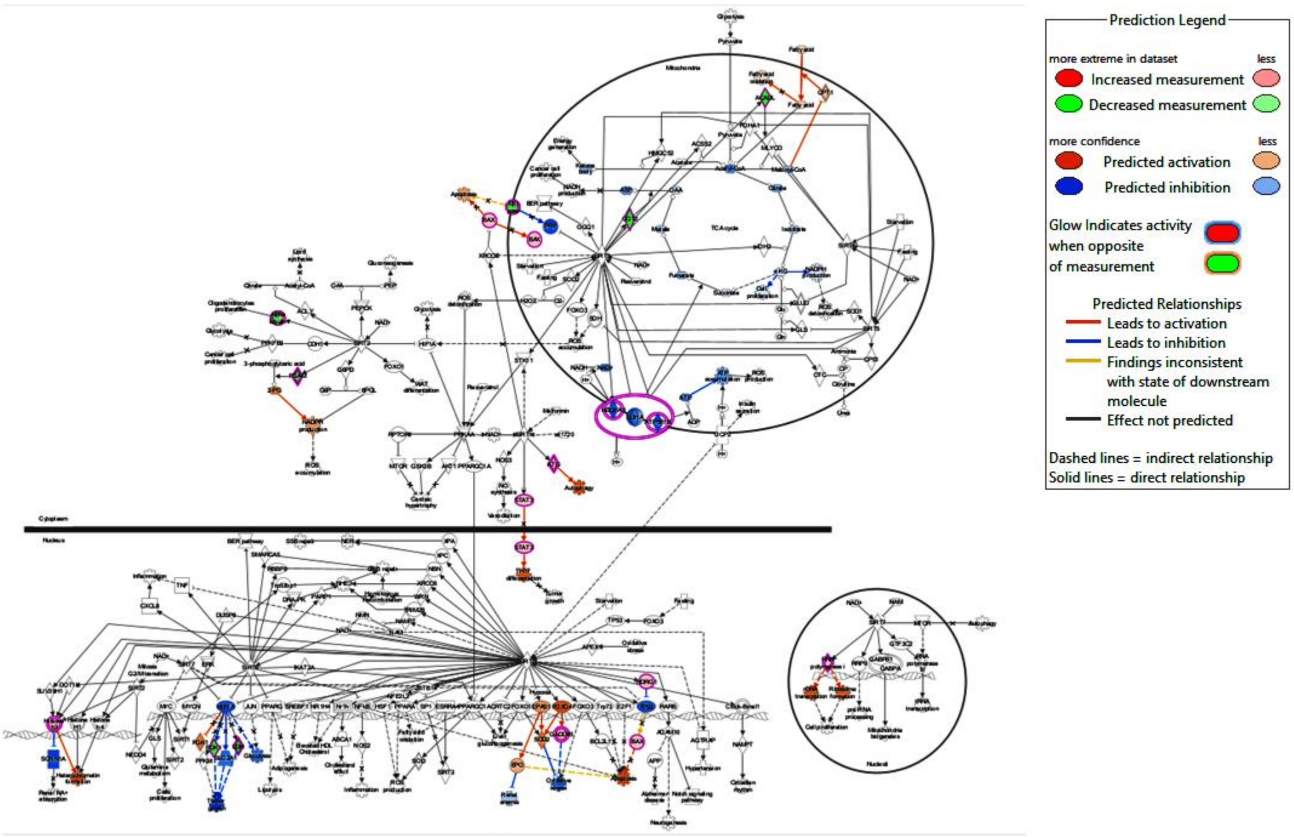

Figure S3: IPA prediction of sirtuin signaling in HFpEF (LS vs HS-sham)

Predicted activation of sirtuin signaling is associated with reduced fatty acid oxidation, reduced generation of acetyl CoA and TCA cycle intermediates, inhibition of mitochondrial oxidative phosphorylation, and reduced ATP generation.

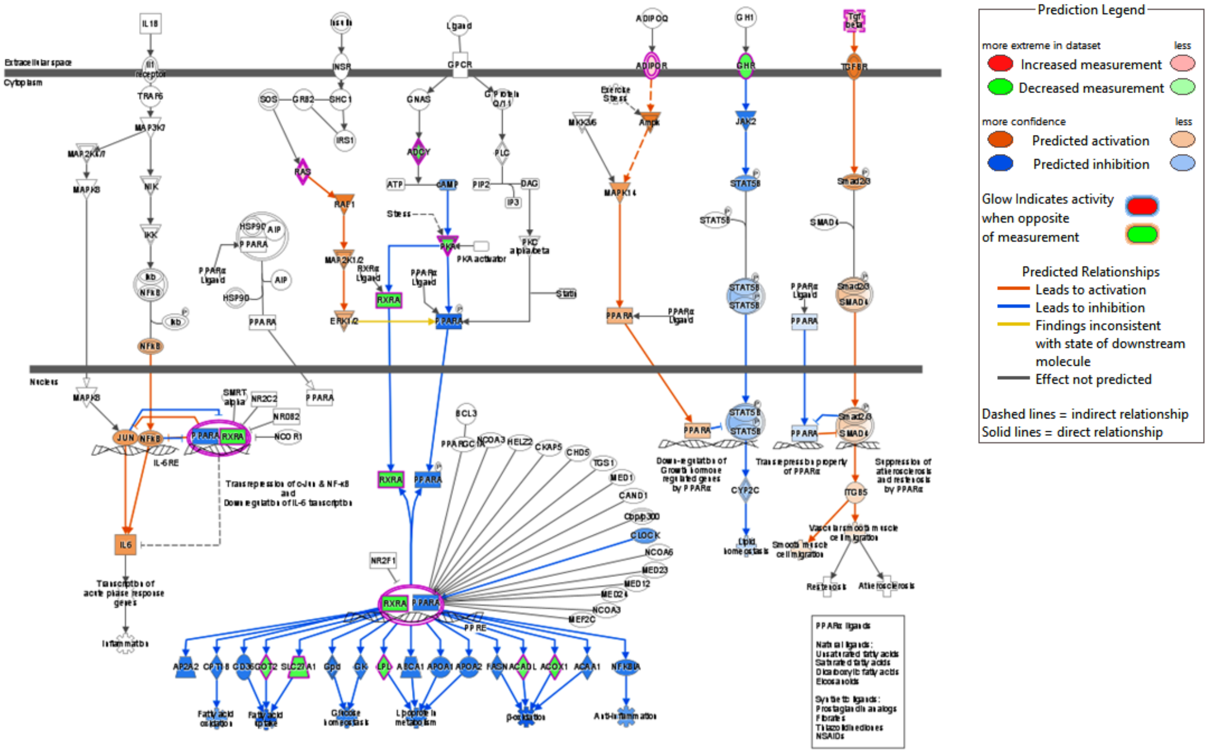

Figure S4: IPA prediction of PPARα/RXRα signaling in HFpEF (LS vs HS-sham)

Predicted inhibition of PPARα/RXRα signaling is associated with the inhibition of fatty acid oxidation and abnormal glucose homeostasis
